# CHRONIC PAIN AND CIRCUMSTANCES OF FALLS IN COMMUNITY-LIVING OLDER ADULTS

**DOI:** 10.1093/geroni/igac059.143

**Published:** 2022-12-20

**Authors:** Yurun Cai, Suzanne Leveille, Ling Shi, Ping Chen, Tongjian You

**Affiliations:** University of Pittsburgh, Pittsburgh, Pennsylvania, United States; University of Massachusetts Boston, Boston, Massachusetts, United States; University of Massachusetts Boston, Boston, Massachusetts, United States; University of Massachusetts Boston, Boston, Massachusetts, United States; University of Massachusetts Boston, Boston, Massachusetts, United States

## Abstract

Chronic pain is a risk factor contributing to mobility impairment and falls in older adults. Little is known about the patterns of fall circumstances among older adults with pain. This prospective cohort study described frequencies of fall circumstances (i.e., location, activities, and self-reported causes of falls) and examined the relationship between chronic pain and fall circumstances among 765 community-dwelling older adults (mean age=78.1, 63.9% women) in the MOBILIZE Boston Study. Pain severity, fall occurrence, and fall circumstances were recorded using monthly calendar postcards and fall follow-up interviews during a 4-year follow-up. Descriptive analyses summarized frequencies of fall circumstances. Generalized estimating equation (GEE) models examined the relation between monthly pain ratings and circumstances of the first fall in the subsequent month. Among 1,829 falls, 965 (52.8%) falls occurred indoors and 804 (44.0%) falls occurred outdoors, 60 (3.2%) falls with missing location information. Commonly reported activities and causes of falls were walking (915, 50.0%), slips/trips (943, 51.6%), and inappropriate footwear (444, 24.3%). GEE models suggested that compared to fallers without pain, fallers with moderate-to-severe pain had around twice the likelihood of reporting indoor falls (adj.OR=1.93, 95%CI:1.32-2.83), falls in living/dining rooms (adj.OR=2.06, 95%CI:1.27-3.36), and falls due to health problems (adj.OR=2.08, 95%CI:1.16-3.74) or feeling dizzy/faint (adj.OR=2.10, 95%CI:1.08-4.11), but they were less likely to report falls while going down stairs (adj.OR=0.48, 95%CI:0.27-0.87) or falls due to slips/trips (adj.OR=0.67, 95%CI:0.47-0.95) in the subsequent month. Future studies may investigate whether better pain management and tailored fall prevention in elders with chronic pain could lead to fewer falls.

